# Correlation Spectroscopy and Molecular Dynamics Simulations to Study the Structural Features of Proteins

**DOI:** 10.1371/journal.pone.0064840

**Published:** 2013-06-04

**Authors:** Antonio Varriale, Anna Marabotti, Giampiero Mei, Maria Staiano, Sabato D’Auria

**Affiliations:** 1 Laboratory for Molecular Sensing, IBP-CNR, Naples, Italy; 2 Department of Chemistry and Biology, University of Salerno, Fisciano (SA), Italy; 3 Laboratory for Bioinformatics, ISA-CNR, Avellino, Italy; 4 Department of Experimental Medicine and Biochemical Sciences, University of Roma “Tor Vergata”, Rome, Italy; Instituto Tecnologia Quimica e Biologica; Universidade Nova de Lisboa, Portugal

## Abstract

In this work, we used a combination of fluorescence correlation spectroscopy (FCS) and molecular dynamics (MD) simulation methodologies to acquire structural information on pH-induced unfolding of the maltotriose-binding protein from *Thermus thermophilus* (MalE2). FCS has emerged as a powerful technique for characterizing the dynamics of molecules and it is, in fact, used to study molecular diffusion on timescale of microsecond and longer. Our results showed that keeping temperature constant, the protein diffusion coefficient decreased from 84±4 µm^2^/s to 44±3 µm^2^/s when pH was changed from 7.0 to 4.0. An even more marked decrease of the MalE2 diffusion coefficient (31±3 µm^2^/s) was registered when pH was raised from 7.0 to 10.0. According to the size of MalE2 (a monomeric protein with a molecular weight of 43 kDa) as well as of its globular native shape, the values of 44 µm^2^/s and 31 µm^2^/s could be ascribed to deformations of the protein structure, which enhances its propensity to form aggregates at extreme pH values. The obtained fluorescence correlation data, corroborated by circular dichroism, fluorescence emission and light-scattering experiments, are discussed together with the MD simulations results.

## Introduction

Fluorescence correlation spectroscopy (FCS) has emerged as a powerful technique for characterizing the dynamics of molecules of biochemical and biophysical interest. Although commonly used to measure the diffusion-associated properties of biomolecules, FCS can also characterize fluctuations in fluorescence intensity caused by chemical kinetics or photo-physics [1]–[4]. In addition, FCS has recently been used as a sensing methodology for the detection of analytes in biomedicine and food safety [5], [6].

A number of studies have demonstrated the advantages of FCS in monitoring both folding and functional properties related to conformational fluctuations in RNA [7], [8], DNA [9], [10], polypeptides [11], and proteins [12]. The timescale of protein conformational fluctuations ranges from picoseconds to seconds, due to the large set of local and global structural changes that might take place in their tertiary structure, even at thermodynamic equilibrium. Several spectroscopic techniques, including steady-state fluorescence spectroscopy and fluorescence dynamics (time and frequency domain lifetime measurements), provide data both in the millisecond and nanosecond timescale, respectively, giving information, for instance, on the dynamics of proteins domains. FCS is particularly suitable to study those protein dynamic features that occur in the intermediate time scale (i.e. the microsecond range) and, consequently, that are hardly detectable by conventional spectroscopic techniques. FCS methodology is based on fluorescence fluctuations that take place in a very small observation volume, typically a few femto-liters. These fluctuations may result either from a change in the number of fluorophores in the observation volume due to diffusion, or from a change in the fluorescence properties of the molecule as a consequence of a chemical reaction or a conformational fluctuation. In the case of proteins, these conformational transitions take generally place in a timescale of microseconds or longer [13].

An additional interesting feature of FCS is the possibility of mapping the folding events of a single molecule at a time. In particular, this approach enables the measurement of intra-molecular diffusion coefficients in denatured and partially protein folded states, providing detailed insights into the nature of the polypeptide chain at different stages of its folding.

A different experimental approach to follow the conformational dynamics of a protein is the *in-silico* simulation via the molecular dynamics (MD) methods. Despite the different timescale investigated (in the range of nanosecond) MD technique is able to identify the first signal of the variation of protein structure associated to the perturbation applied to the system. MD offers the opportunity to obtain an analysis at molecular level of the protein structural variations. In the past, we have already successfully combined the spectroscopic and computational approaches to study the features of several proteins in different conditions of pH and temperature [14]–[16]. We were able to match information from both approaches providing a more complete portrait of the phenomena affecting the bio-molecules.

In the present study the advantage is even greater as both investigation techniques are focused on the study of a single molecule, and therefore the comparison of the results is straightforward.

Hence, we have explored the possibility of using FCS as a tool to measure conformational dynamics and diffusion properties of the maltotriose-binding protein (MalE2) isolated from *Thermus thermophilus* in its folded, unfolded and intermediate states as induced by different pH values. The gene of MalE2 was isolated and identified by Silva et al. [17]. A preliminary characterization of MalE2 has showed that it is a monomeric protein with a molecular weight of 43 kDa that exhibits an extremely high thermal stability, having a melting temperature at 105°C in the presence of 2.3 M of guanidinium chloride [17]. Recently, the structure and some functional features of MalE2 have been clarified. In fact, the X-ray crystal structure of MalE2 in complex with maltotriose has been resolved at 1.95 Å resolution [18], and binding studies have showed that MalE2 binds to the tri-saccharide maltotriose with a high affinity.

In the present study MalE2 protein was labeled with the fluorescent probe Alexa Fluor 488 (AL488) and FCS measurements were carried out in solutions ranging from pH 2.0 to pH 11.0 keeping the temperature at 25°C. MD simulations were applied to the model of un-liganded form of MalE2, in order to elucidate at molecular level the impact of pH on protein structure. From the experimental and *in-silico* results, a protein portrait has been depicted showing that at acidic pH values the protein undergoes to a conformational transition towards different molecular forms through the formation of intermediate species. This transition seems to affect mainly the tertiary structure of the protein suggesting the formation of an intermediate state that evolves into a protein unfolded form. At basic pH values, instead, the diffusion coefficient of the protein slightly decreases to the value exhibited at neutral pH values. The spectroscopic data are discussed together with the MD simulations results.

## Materials and Methods

### Reagents

Standard chemicals, solvents, and buffers were purchased from Sigma-Aldrich. Alexa Fluor 488 (AL488) was purchased from Molecular Probes, Invitrogen (Eugene, OR, USA).

### MalE2 Purification

BL21 Rosetta strain, containing pTRCGE-MalE2, was cultivated at 37°C in Luria-Bertani (LB) medium containing 100 µg/mL ampicillin and 50 µg/mL chloramphenicol [17]. Strain DH5, containing pTRCGT-MalE2, was cultivated in the same medium with 100 µg/mL ampicillin. When an OD^610^ of 1.0 was reached, the expression of GST-MalE2 fusion proteins was induced by adding 0.5 mM IPTG (isopropyl-D-thio-galacto-pyranoside). After 3 h of incubation, the cells were harvested by centrifugation (7,000 g, 10 min, 48°C); suspended in phosphate-buffered saline at pH 7.3, containing DNAse I (10 µg per milliliter of suspension) and the protease inhibitors phenyl-methyl-sulfonyl fluoride (80 µg per milliliter of suspension), and ruptured by sonication. To remove cell debris, the extracts were centrifuged (18,000 g, 1 h, 48°C) and filtered through 0.22 µm-pore-size-filters (Schleicher & Schuell). The extracts were applied to a GSTprepFF16/10 column and eluted with 50 mM Tris-HCl and 10 mM glutathione pH 8.0 (Sigma). The fractions containing MalE2 were treated with enterokinase (Novagen) or thrombin (Amersham Biosciences), respectively, for 16 h at 24°C. To separate GST from MalE2, the samples were applied to a Mono-Q fast-flow column equilibrated with 20 mM Tris-HCl pH 7.6. Elution was carried out with a linear NaCl gradient (0.0 M to 1.0 M). Those fractions contained MalE2 were concentrated, and the purity of the samples was evaluated by sodium dodecyl sulfate-polyacrylamide gel electrophoresis (SDS-PAGE).

### Protein Assay

The protein concentration was determined by the method of Bradford [18] with bovine serum albumin as standard on a double beam Cary 1E spectrophotometer (Varian, Mulgrade, Victoria, Australia).

### MalE2 Labeling

A solution of MalE2 at concentration of 1.0 mg/mL in 1.0 mL was dissolved in 0.1 M sodium bicarbonate buffer, pH 7.0 and mixed with AL488. The molar ratio of the dye and the protein was kept 10:1. The reaction mixture was incubated for 1 h at room temperature and the labeled molecules were separated from un-reacted probe by gel filtration and dialysis procedure against 50 mM phosphate buffer pH 7.0 by using dialysis tubes with a cutoff of 3500 Da (Spectrum Labs) overnight, at 4.0°C. The extent of labeling was calculated to be approximately 89%.

### pH Effect

pH effect was studied in the range 2.0–11.0. The FCS measurements at different pH values were carried out in 10 mM solutions of the following buffers: glycine/HCl (pH 2.0–3.0), sodium acetate (pH 4.0–6.0), sodium phosphate (pH 7.0–8.0), and sodium carbonate (pH 9.0–11.0). All the solutions were prepared in distilled water and the MalE2-AL488 samples, at different pH values, were prepared by dissolving the protein at the final concentration of 10.0 nM.

### Fluorescence Correlation Spectroscopy Measurements

Before making FCS measurements the solutions were incubated overnight at 4.0°C. All FCS experiments were carried out at room temperature. Experiments were performed with the ISS-ALBA (Urbana-Champaign, IL, U.S.A.) fluorescence correlation spectrometer equipped with a Nikon inverted microscope. Two-photon excitation (in the range 780–800 nm) were provided by a Ti:sapphire mode-locked laser (Chameleon Ultra; Coherent Inc, Santa Clara, CA, USA). The instrument alignment was performed using a dilute solution (10.0 nM) of rhodamine 110. At each pH value a preliminary measurement with rhodamine, at a known concentration (8.0 nM), was carried out in order to evaluate the excitation volume to be used in the data analysis. All pinhole adjustments, shutters, optics, filter wheels, XYZ-fine positioning of the stage and the positioning of the objective are computer controlled through Vista, the FCS software package.

### FCS Data Analysis

The obtained experimental correlation function were fit to the equation appropriate for 3D Gaussian beam shape using Vinci Analysis program (ISS inc., Urbana, IL) and in particular with the following equation:
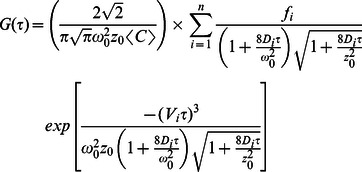
(1)


In this equation, G(τ) represents the autocorrelation function, D is the diffusion coefficient of the molecules, ω_0_ and z_0_ are the radial and axial dimension for a three-dimensional Gaussian observation volume and C is the molecular concentration.

The diffusion coefficient D of a molecule can be calculated from τ using Equation 2:
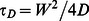
(2)where W is the beam radius of the observation volume, which can be obtained by measuring τ_D_ of a fluorophore of known diffusion coefficient D.

Before the measurements were done, the observed volume was calibrated with a solution of 8.0 nM rhodamine 110 using the known diffusion coefficient of 300 µm^2^/s [20]. Also the diffusion times of individual molecules (Rhodamine 6G, glutamine-binding protein, bovine serum albumin) were measured, and these values were used as fixed parameters. Also different experimental controls were carried out in the FCS measurements to be sure that the external exponential component, obtained with Equation 1, had been calculated for the motion of the protein and not for the experimental artifacts. As it has been demonstrated in literature [21] one possible artifact could be the presence of free dye in the solution, therefore gel filtration and extensive dialysis were performed to remove the un-react probe before the FCS measurements were made. Another artifact could be that the observed volume does not have a Gaussian shape, therefore using the two photon excitation setup we were sure that the Gaussian shape of the observed volume is maintained over all measurements.Also the hydrodynamic size of MalE2 was calculated using the spherical Stokes-Einstein approximation:
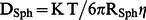
(3)or alternatively using the Perrin prolate ellipsoidal model (relative to a sphere of equal volume):
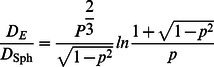
(4)where η, the viscosity of water at 25°C, is 0.89 cP (1 P = 0.1 Pa s), K is the Boltzmann’s constant, T is the temperature and p = b/a is the ratio of the prolate ellipsoid semi-axes.

### Circular Dichroism Spectroscopy

Circular dichroism (CD) spectroscopy was performed on homogeneous samples of MalE2 at concentration between 0.2 and 0.5 mg/mL in 2.0 mM sodium phosphate buffer, pH 7.0, 2.0 mM sodium acetate buffer pH 4.0 and 2.0 mM sodium carbonate buffer at pH 10.0. Jasco J-810 spectropolarimeter (Jasco, Tokyo, Japan) equipped with the Neslab RTE-110 temperature-controlled liquid system (Neslab Instruments, Portsmouth, NH, USA) and calibrated with a standard solution of (+)-l-O-camphorsulfonic acid was used for the measurements. Sealed cuvettes with a 0.1 cm and 1.0 cm path length (Helma, Jamaica, NJ, USA) were used in the far and near-UV region, respectively. Photomultiplier high voltage did not exceed 600 V in the spectral regions measured. Each spectrum was averaged five times and smoothed with Spectropolarimeter System Software (Jasco, Japan).

### Steady State and Dynamic Fluorescence Measurements

Steady-state fluorescence spectra were recorded on a K2-ISS fluorometer (ISS, Champaign, IL, USA). Excitation was set at 295 nm in order to exclude the tyrosine contribution in MalE2 bulk fluorescence. The emission data were collected from 310 to 410 nm.

MalE2 at different values of pH was incubated with 10-fold molar excess of 8-anilinonaphthalene-1-sulfonic acid (ANS) for 30 minutes at room temperature in dark condition. Then, fluorescence emission spectra were measured in the range 400–580 nm with λ_exc_ = 370 nm.

Dynamic fluorescence measurements of MalE2 at pH 4.0, pH 7.0 and pH 10.0, were performed with a KOALA-ISS fluorometer, (ISS, Champaign, IL, USA) using the phase shift and demodulation technique. The excitation source (295 nm) was a LED; emission was collected through a 320 WG cut-off filter to avoid scattered light. As lifetime reference was used the 2,5-diphenil-1,3,4-oxidiazole and the data were fitted according to a double lorentzian-shaped continuous distribution of lifetime.

### Dynamic Light Scattering Measurements

Light scattering measurements were performed on a Horiba (Kyoto, Japan) LB-500 dynamic light scattering nanoparticle size analyzer, equipped with a 650 nm, 5 mW laser diode. Each measurement has been repeated 5–6 times in order to get a better statistics on the average value. Data analysis was performed using the accompanying software based on a Fourier-transform deconvolution procedure.

### Modelling of the Structure of MalE2

The open un-liganded form of MalE2 was modeled using as template the open un-liganded form of the maltotriose binding protein from *T. maritima* (PDB code: 2GHB) available in PDB archive [22], found with a BLAST [23] search against PDB database. In order to optimize the creation of the model exploiting also information from the crystallographic structure of the close form of MalE2 with maltotriose (PDB code: 2GH9) [18], a procedure similar to that we applied previously on ArgBP was applied [24]. Briefly, the protein from *T. maritima* was used as a “scaffold” to create a “hybrid” structure in which the two domains of MalE2 are oriented as in the open un-liganded form and the connection between the two domains is made by the protein from *T. maritima*. Using this “hybrid” structure as template, 10 models of the complete structure of MalE2 were obtained using Modeller 9v7 [25]. The model chosen among those created by Modeller was evaluated for its stereochemistry using PROCHECK [26] and for its energy using Prosa II [27].

### Molecular Dynamics Simulations

The model of open un-liganded form of MalE2 was used to perform MD simulations experiments at room temperature (27°C) and at three different pH values: pH 4.0, pH 7.0 and pH 10.0. The program GROMACS version 4.0.7 [28], [29] was used running in parallel (MPI) on the supercomputer “CRESCO”, formed by 300 nodes in SMP (total number of cores: 2700) and 34 multiprocessor servers for specific functions, all interconnected each others by an Infiniband net. The GROMOS96 force field [30] was used throughout the simulations. The protein was included in a triclinic box with a distance from solute of 1 nm per side, filled with water molecules (SPC model) [31]. To simulate the different pH, a preliminary analysis was performed of the pKa of the charged residues Glu, Asp, His, Lys and Arg in the protein, using the Web server PROPKA 3.0 [32]. Then, three different systems were created in which the charged groups were protonated or un-protonated according to different pH, using as reference their pKa. In the system at pH 4.0, one His is positively charged, 8 Asp and 4 Glu residues are negatively charged and all Lys and Arg residues are protonated; in the one at pH 7.0 all His are neutral, all Asp and Glu residues are negatively charged and all Lys and Arg residues are protonated; finally, at pH 10.0 all Asp and Glu residues are deprotonated and Arg residues are protonated, and 3 Lys residues are deprotonated. 32 and 5 Cl^-^ ions, respectively for systems at pH 4.0 and 7.0, and 4 Na^+^ ions for the system at pH 10 were added to the solvent to neutralize the net charge of the whole system, replacing the corresponding number of water molecules, with the aid of GROMACS utilities. Periodic boundary conditions were used to exclude surface effects.

For the three systems, a preliminary energy minimization step with a tolerance of 500 kJ/mol/nm was run with the Steepest Descent method. All bonds were constrained using P-LINCS [33]. After minimization, a short NVT (fixed volume and temperature ) MD simulation (20 psec) with position restraints was applied to each system to soak the solvent into the macromolecule. A time step of 2 fs was used in all cases, and the systems were coupled to a temperature bath at room temperature using V-rescale, a thermostat that uses velocity rescaling with a stochastic term [34]. Long-range electrostatics were handled using the PME method [35]. Cut-off were set at 1.0 nm for Coulomb interactions, and at 1.4 nm for van der Waals interactions. This short MD was followed by another short (100 ps) MD in NPT conditions (fixed temperature and pressure). A pressure of 1 bar was coupled using the Berendsen’s method [36]. Finally, a 50 ns-long simulation was performed separately for each pH condition, with a time-step of 2.0 fs and without any position restraints. All other conditions were kept identical as the short NPT dynamics.

At the end of simulations, the energy components were extracted from the energy files generated by the program, and analyzed to verify the stabilization of the system. After that, several analyses were conducted using programs built within GROMACS, and results were visualized and elaborated with the aid of the freely available program Grace (http://plasma-gate.weizmann.ac.il/Grace). The cluster analysis was made using the clustering method of Daura [37], with a cut-off of 0.25 nm. Principal component analysis (PCA) [38] was also performed on the trajectories. The covariance matrix was built from the position coordinates of Cα atoms. The results were used to represent the free energy landscape of the systems in different conditions, calculating the probability of finding the system in a particular state, as a function of the free energy state [39]. Visual inspection of the structures and pictures were carried out with the Insight II package (Version 2000.1, Accelrys, Inc.; 2000).

## Results and Discussion

### Fluorescence Correlation Spectroscopy

In this work we have studied the pH dependence on the conformational dynamics and diffusional properties of protein MalE2, isolated from *Thermus thermophilus*. In particular, we have investigated the MalE2 folds, unfolds and intermediate unfolded protein states induced by different pH values keeping constant temperature at 25°C.

The FCS measurements at different pH values and 25°C were carried out to monitor the protein unfolding transitions and were performed on amino terminus Alexa-labeled MalE2 (MalE2-AL488) in the pH range 2.0–11.0.

**Figure 1:**
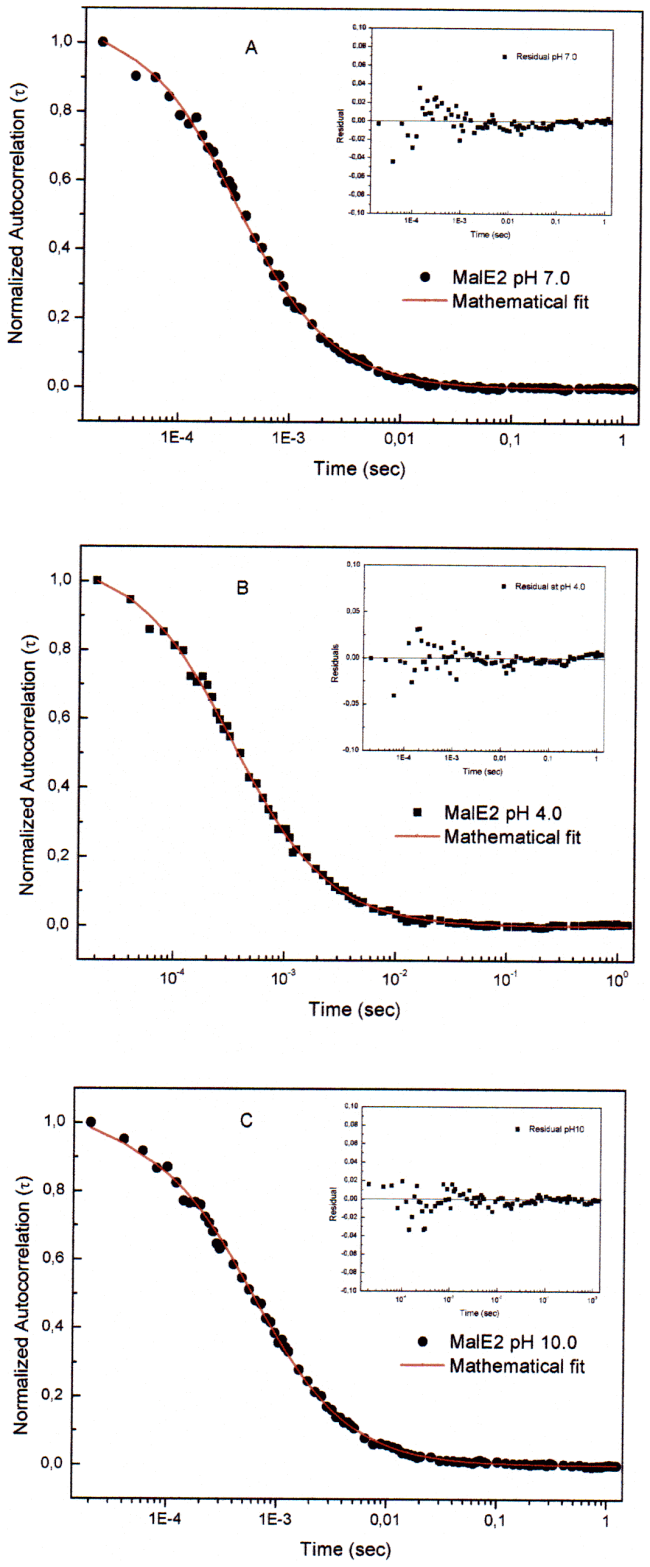
Fluorescence correlation spectroscopy measurements. Autocorrelation spectra and residual distribution (inset) of MalE2 at pH 7.0 (panel A), pH 4.0 (panel B) and pH 10.0 (panel C). The best fitting of the data was obtained by the Equation 1.

The correlation function observed in the FCS measurements on MalE2-AL488 in its native state at neutral pH (panel 1A) and at pH 4.0 (panel 1B ) and 10.0 (panel 1C) is shown in Figure 1. The data were analyzed by different models and the best fitting function resulted to be Equation 1, which takes into account the possibility to have one or two species in solution. In particular, the MalE2-AL488 diffusion coefficient at pH 7 was found to be D_1_ = 84±4 µm^2^/s, a value compatible with a spherical-shaped molecule with a radius of about 29 Å (Table 1). In these conditions, no improvement of the quality of the fit was observed introducing a second diffusing species in the fitting function (Table 1). The measurements at pH 4.0 and pH 10.0 yielded diffusion coefficient values of 44±3 µm^2^/s and 31±6 µm^2^/s, respectively (Table 1) that can be explained in terms of ellipsoidal shaped protein molecules. In these cases, a small amount (2–3%) of slower diffusing molecules (D_2_ = 15 µm^2^/s) resulted also to be present (Table 1), suggesting that the protein might form aggregates.

**Table 1:** Diffusion coefficients obtained by FCS measurements (Figure 2) and geometrical properties of MalE2 molecule (see Materials and Methods section for more details).

	?2(1)	D_1_ (µm^2^/s)	F_1_ (%)	D_2_(µm^2^/s)	(2)R_Sph_(Å)	(3)a(Å)	(3) b(Å)	DE(µm^2^/s)
pH 4.0 (1 species)	0.8	44±3	100	–	57±8	34.8	20.8	43
pH 4.0 (2 species)	0.7	43±3	97	14±3				
pH 7.0 (1 species)	0.7	84±4	100	–	29±3			
pH 7.0 (2 species)	0.7	83±4	99	15±3				
pH 10.0 (1 species)	1.1	31±6	100	–	81±3	46.8	19.2	30
pH 10.0 (2 species)	0.8	30±5	98	13±3				

(1) Errors on FCS measurements**.**

(2) Molecular radius (R_Sph_ calculated from the D_1_ diffusion coefficient using equation 3).

(3) Prolate ellipsoid semi-axes (a, b) and corresponding diffusion coefficient (DE) calculated using Equation 4, assuming the same spherical volume obtained at pH 7.0 (i.e. ≈ 1·10^5^ Å^3^).

**Figure 2:**
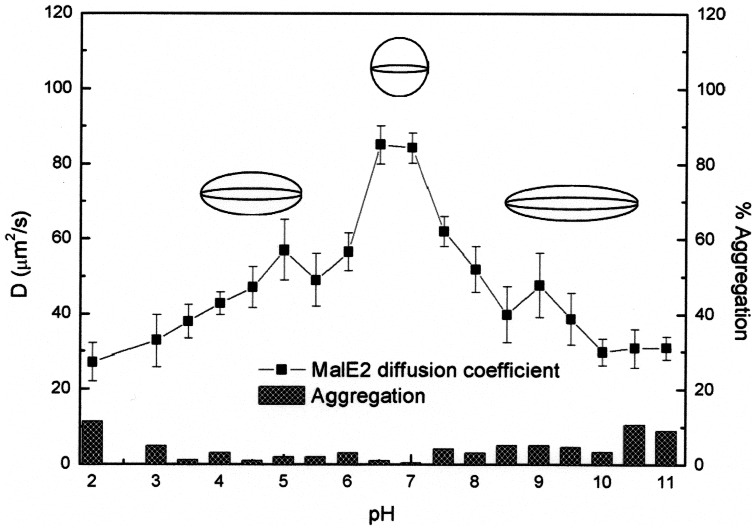
Dependence of diffusion coefficient (D) as a function of pH. The error bars of the measurements were obtained by using three different measurements. The histogram shows the aggregation percentage (axis on the right) as a function of pH values. The sphere and the two ellipsoids represent (in scale) the possible shapes of MalE2 (at pH 7.0, pH 4.0 and pH 10.0) compatible with the corresponding diffusion coefficients measured by FCS (see Table 1 and Materials and Methods section).

The pH dependence of MalE2-AL488 diffusion coefficient, D_1_, is shown in Figure 2. The data indicate a complex behavior consisting in several distinct steps. In the initial part of the titration curve (between pH 2.0 and pH 4.5) the value of D progressively increases from 25 to 50 µm^2^/s, then remaining constant up to pH value 6.0. This result suggests the presence of intermediate and stable protein species. The second part of the titration curve (from pH 6.0 to pH 7.0) is characterized by a steeper increase of the diffusion coefficient and is followed by the last step (in the range pH 7.0–10.0), in which D decreases to smaller values, within 30–40 µm^2^/s.

In Figure 2 the percentage of slowly diffusing molecules is also reported at each pH value, as extrapolated from the two components fit of the FCS data. The fractional contribution of this second component remains below 5% in almost all the pH range explored (pH 3–10), reaching instead 10% at extreme basic or acid conditions. Such behavior indicates that the protein propensity to form aggregates is particularly enhanced at extreme pH values. These data are in good agreement with the light scattering results (see below).

### Hydrodynamic Radius of MalE2

According to the Stokes-Einstein equation (Equation 3), we estimated the hydrodynamic radius (R_Sph_) of MalE2-AL488 from the diffusion coefficient values calculated by analyzing the FCS data. The data indicate that at neutral pH the hydrodynamic radius is R_Sph_ = 29±3 Å. Recently, several studies have reported the values of the hydrodynamic radii of proteins calculated from FCS data in native and denaturant conditions (pH, temperature, and in the presence of chemical compounds e.g. guanidine chloride) [40]. The obtained R_Sph_ values resulted to be in good agreement with the calculations proposed by Wilkins and collaborators [41] on the basis of NMR diffusing measurements of a large number of proteins. According to this empirical approach, the dependence of R_Sph_ on the total number of residues for native proteins is R_N_ =  (4.75±0.15) N^0.29±0.02^. When we applied this equation to the case of MalE2 we obtained R_N_ = 26.5±0.8 Å, and this value corresponds, within the experimental error, to that obtained from the diffusion coefficient measured by FCS (Table 1).

**Figure 3:**
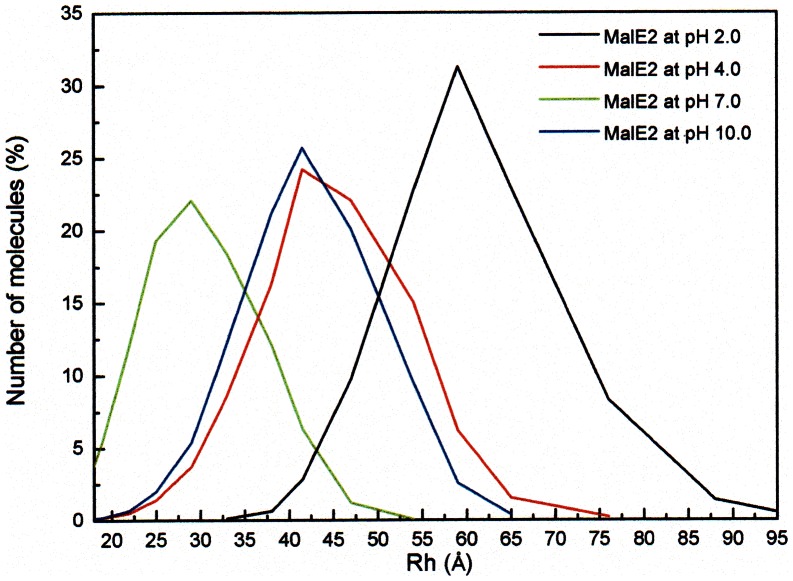
Dynamic light scattering measurements. Particle size distribution as a function of pH of MalE2 at pH 2.0 (black), pH 4.0 (red), pH 7.0 (green) and pH 10.0 (blue). The measurements were performed at 25°C. The final concentration of protein was 0.1 mg/ml.

Light scattering measurements (Figure 3) also indicate that at pH 7.0 the distribution of the MalE2 molecules radius in solution is centered around 28 Å, confirming that the Stokes-Einstein model is a good approximation in the case of the native protein. On the contrary, using the diffusion coefficients obtained both in acid (pH 4.0) and basic (pH 10.0) conditions yielded (through equation 3) unreliable R_Sph_ values (57 and 81 Å, respectively), much larger than those observed in the light scattering measurements (Figure 3). Such a discrepancy suggests that: i) in these conditions the system is probably more heterogeneous than at pH 7.0, and ii) a different model must be applied to explain the decrease of the D_1_ value. The first hypothesis is supported by both light scattering (Figure 3) and FCS (Figure 2) measurements, which indicate a progressive increase in the protein aggregation propensity, as the pH is changed from pH 7.0 to pH 2.0 or pH 10.0.

On the other hand, we have used the Perrin diffusional theory of ellipsoidal particles [42] to check whether the D_1_ diffusion coefficients measured by FCS at pH 4.0 and pH 10.0 are compatible with changes in the shape of the MalE2 molecule. As a round down approximation, we imposed to a prolate ellipsoid model of MalE2 the same volume of the native protein (i.e. ≈ 100000 Å^3^) and the resulting semi-axes values, using the equation 4, are reported in Table 1. The results suggest that the decreased diffusion capability of MalE2 might indeed arise from pH-induced change in the protein shape, as schematically reported in Figure 2.

### CD and Fluorescence Spectroscopy Analyses

**Figure 4:**
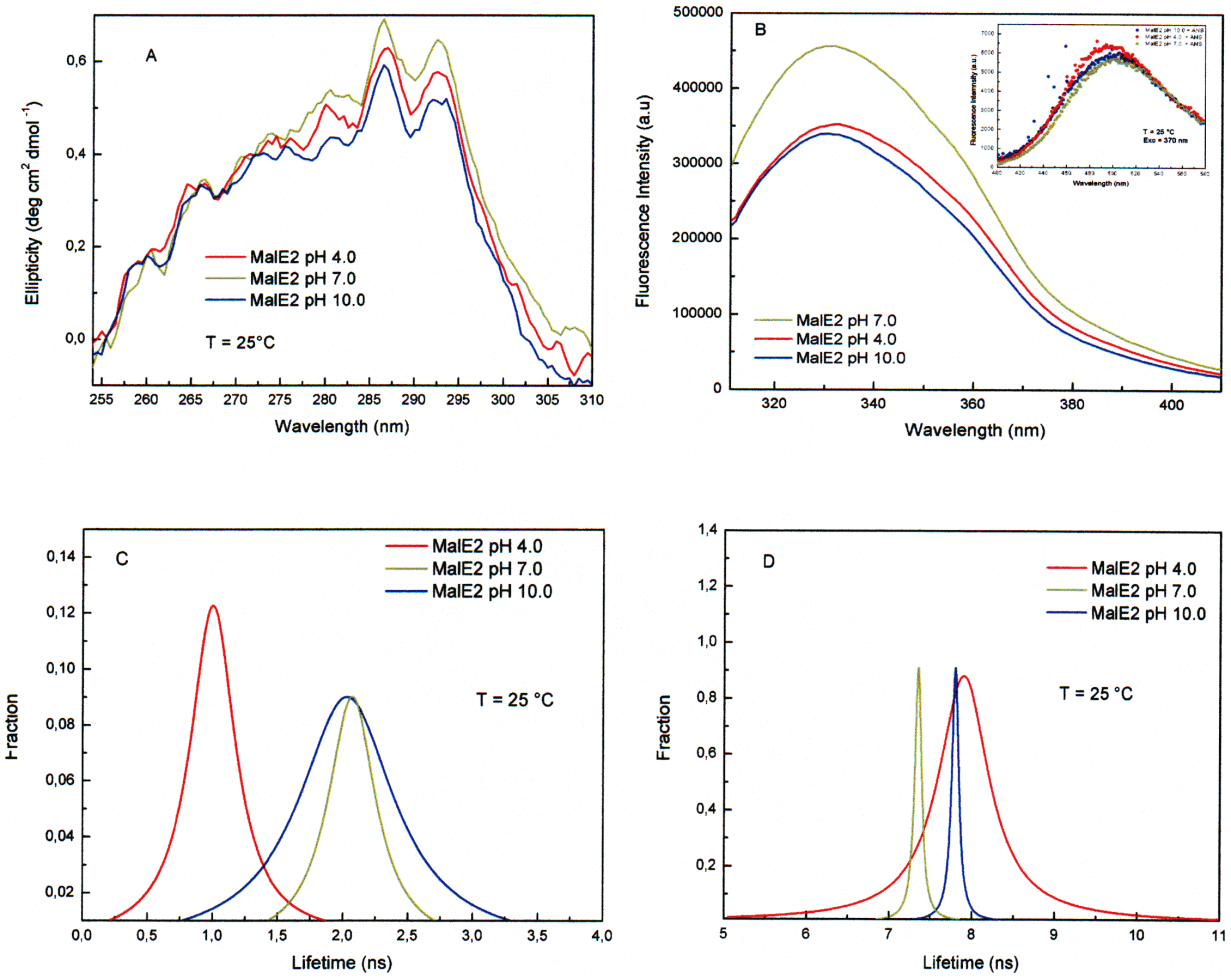
Circular dichroism and fluorescence measurements. Panel A: Near-UV spectra of MalE2 at pH 4.0 (red), pH 7.0 (green) and pH 10.0 (blue); Panel B: steady state fluorescence spectra of MalE2 at pH 4.0 (red), pH 7.0 (green) and pH 10.0 (blue); inset: the ANS fluorescence spectra of MalE2 at pH 4.0 (red), pH 7.0 (green) and pH 10.0 (blue); Panel C and D: lifetime distribution profiles obtained from dynamics fluorescence measurements at pH 4.0 (red), pH 7.0 (green) and pH 10.0 (blue).

Circular dichroism measurements in the far-UV yielded superimposable spectra of MalE2 at pH 7.0, pH 4.0 and 10.0, at 25°C (data not shown), indicating that alkaline and acidic pH do not have a measurable effect on the secondary structure content of MalE2. A similar result was almost expected, since also the secondary structure content of other α/βproteins resulted to be independent on pH variations [43], [44]. On the contrary, CD measurements in the aromatic region (250–300 nm) revealed that detectable spectral differences arise when the pH value is varied from 4.0 to 10.0 (Figure 4A). In particular, a decrease in the intensity of the typical absorption peaks of tryptophan residues (i.e. 280, 285 and 292 nm) is observed both at pH 4.0 and pH 10.0. This result indicates that the protein side chains are indeed more flexible at acid and alkaline pH values, suggesting a larger hydration of the MalE2 tertiary structure at pH 4.0 and pH 10.0 than at pH 7.0.

The effects of pH on the MalE2 structure have been also investigated by monitoring the tryptophan intrinsic fluorescence of MalE2. Steady-state fluorescence measurements were performed on MalE2 at different pH values. The Figure 4B shows the fluorescence emission spectra of MalE2 at pH 7.0, pH 4.0 and pH 10.0 at 25°C. The position of the fluorescence emission maximum is blue-shifted respect to the emission maximum of N-acetyl-tryptophanylamide (NATA) centered at 350 nm (data not shown), suggesting that the tryptophanyl residues of MalE2 for each pH values are in buried and/or un-relaxed microenvironments.

The fluorescence spectra recorded at pH 4.0 and 10.0 are quenched with respect to the spectrum at pH 7.0, suggesting that changes are taking place at the level of the protein tertiary structure as consequence of pH variation.

This hypothesis was further confirmed studying the fluorescence of the ANS-bound protein at different pH values. 1-Anilino-8-naphthalenesulfonic acid (ANS) is a fluorescence dye, whose emission properties undergo detectable changes when ANS binds to the hydrophobic patches of proteins [45]. In particular, in the presence of protein molecules, an increase in the fluorescence intensity and a blue shift of the ANS spectrum are generally indicating the presence of a less compact protein structure. The steady-state fluorescence emission spectra reported in Figure 4B (inset) demonstrate that a loosening of MalE2 tertiary structure occurs when pH is changed from 7.0 to 4.0 or 10.0. In particular, the larger effect is observed at pH 4.0, with a spectral shift of about 10 nm and an increase of 13% of the total fluorescence emission intensity. This finding, which is in line with the CD measurements in the aromatic region, corroborates the occurrence of progressive unfold protein states at extreme pH values.

Finally, the effects of pH on the MalE2 structure have been also investigated by dynamic fluorescence spectroscopy. The dynamic fluorescence of tryptophan residues is, in fact, extremely sensitive to structural changes occurring in the immediate surroundings of the indolic ring, allowing to detect even subtle conformational changes occurring in the tertiary structure of proteins. The tryptophan emission decay properties of MalE2 were measured by frequency domain fluorimetry. The best fit was obtained using a binomial distribution of lifetimes, characterized by a lorentzian shape. The analysis of dynamic fluorescence data in terms of multiple continuous functions is quite common and it has been used in the past to characterize the decay of several, multi-tryptophan containing proteins [46]–[49]. As shown in Figure 4C and 4D, the emission decays of MalE2 are substantially characterized by two components centered around 2.1 and 7.3 ns and displaying different full width at half maximum (0.1 and 0.5 ns, respectively). Such results indicate that the MalE2 tryptophan residues could be grouped in two different classes each of which with different emissive features: one more exposed to the solvent (short-lived component) and the other one being more buried in the protein matrix (long-lived component) [46], [43], [50]. Furthermore, the difference in the width of each protein lifetime distribution suggests that the heterogeneity of microenvironments surrounding the tryptophan residues is different. Both components are strongly influenced by changes in the pH value. In particular, at pH 4.0 the faster lifetimes component is considerably shifted towards shorter values (≈1 ns), suggesting a more efficient quenching effect on the tryptophan residues. The width of the longer component is 7–8 times larger than that obtained at pH 7.0, thus indicating that more buried tryptophan experiences a much larger number of structural conformational sub-states in acid buffer. These findings could be explained in terms of a partial unfolding of the protein tertiary structure at pH 4.0, in line with the results obtained in both CD and ANS-binding measurements. The analysis of the fluorescence dynamics at pH 10.0 demonstrates that also in this case changes occur in the both lifetime components, the main effect being the widening of the short component (Figure 4C).

### Computational Biology Simulations

**Figure 5:**
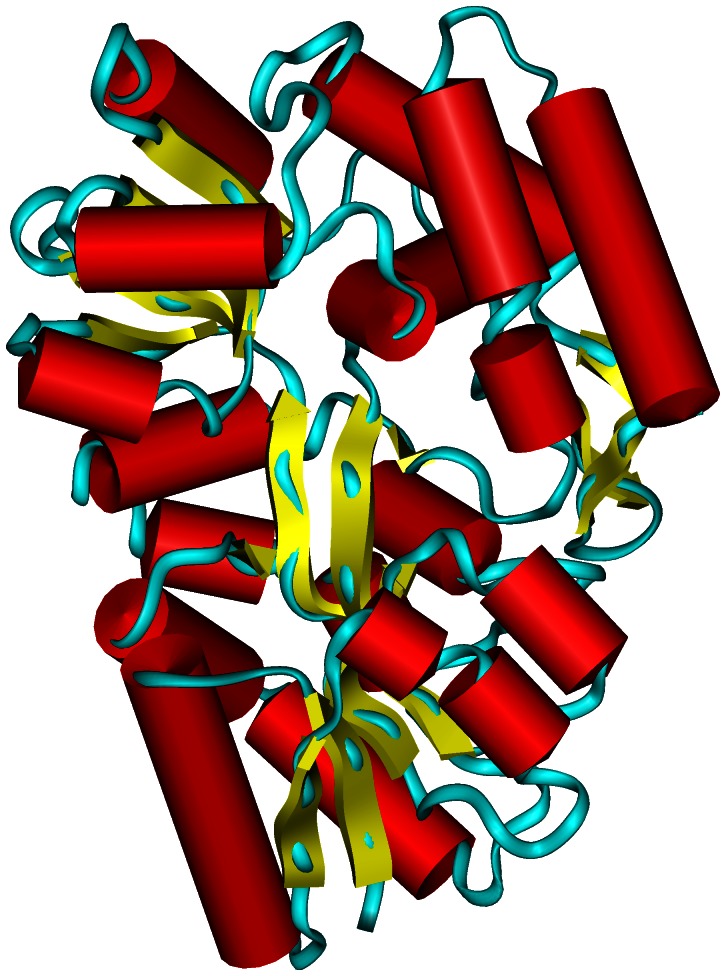
Model of the 3D structure of MalE2 in the un-liganded form. The backbone of the protein is represented as a ribbon, and segments of secondary structures are shown as cylinders (helices) and arrows (strands).

The only available X-ray structure of MalE2 was obtained in complex with maltotriose [18] (PDB code: 2GH9). Unfortunately, this model is not very useful to discuss the observed pH-induced conformational changes in the maltotriose-free enzyme since the proteins belonging to the MalE2 family drastically change their tridimensional structure in the presence of the ligand [51]. We therefore decided to model *in-silico* the open and ligand-free form of MalE2 in order to simulate the behavior of the protein in the correct conformation. This strategy (as described in Material and Methods section) allowed us to obtain a reliable model using as reference the structure of MalE2 itself. We took advantage of the structure of the un-liganded form of the maltotriose-binding protein from *T. maritima* to model the connections between the two protein domains. The reciprocal orientation of the two protein domains in the open form (95.2% of the residues of the selected model) are in the most favored regions of the Ramachandran plot, with no residues in disallowed protein regions (the analysis performed on the template showed 93.7% and 0% of residues for most favored and disallowed regions, respectively). ProsaII z-score (−13.67) is in the range of scores typically found in proteins of similar sequence length [27] and it is similar to that of the template (−14.72). We also analyzed the energetic profile calculated by ProsaII on the whole protein structure, and our selected model displayed an optimal profile, with no positive peaks indicating errors in the structure (data not shown). These data demonstrate that our model is of high quality. Figure 5 shows the model of MalE2 in the protein un-liganded state.

MD simulations of this protein at different pH values were thus performed in order to figure out a possible explanation, at the molecular level, of the results obtained in the FCS measurements (Figure 2). The analysis of the simulations shows that in all cases the energy, the temperature and the pressure of the system are stable along the whole simulations (data not shown). Also the radius of gyration of the protein is stable at about 2.10 nm, with minimal differences among the three different states (namely pH 4.0, 7.0 and 10.0). All these data indicate that a thermodynamic equilibrium has been reached by the system in all states. With the GROMACS tool **g_msd,** it is possible to compute the mean square displacement (MSD) of atoms from their initial positions. This provides an easy way to compute the diffusion constant using the Einstein relation. The diffusion constant is calculated by the program by least squares fitting a straight line through the MSD. We applied this tool, in order to calculate the theoretical diffusion constant of our protein at different pH values. The results for the systems at pH 7.0 and 10.0 (71.8±12.5 µm^2^/sec and 42.3±14.8 µm^2^/sec, respectively) are in excellent agreement with those obtained by experimental approaches, whereas the data at pH 4.0 (12.6±12.9 µm^2^/sec) is meaningless; however the not considering the standard error is in line with the experimental data.

**Figure 6:**
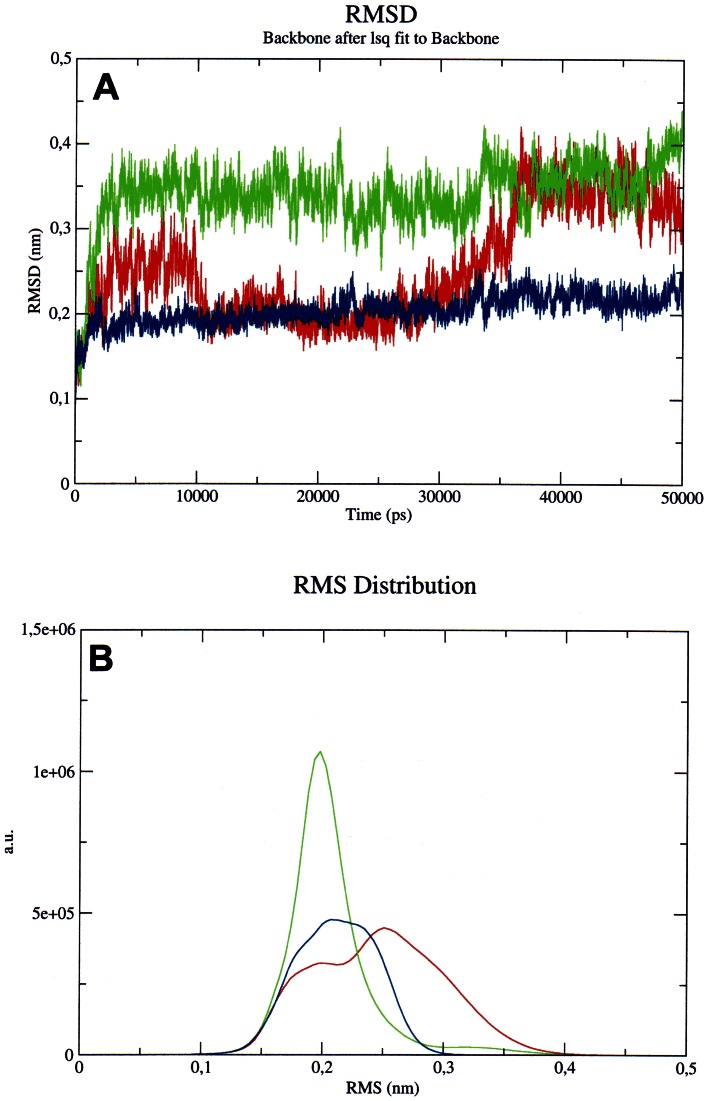
Analysis of RMSD of MalE2. Panel A : RMSD of MalE2 along the simulation, with respect to the starting structures at pH 4.0 (red), pH 7.0 (green) and pH 10.0 (blue). Panel B: RMS distribution of the structures in the clusters calculated along the three simulation at pH 4.0 (red), pH 7.0 (green) and pH 10.0 (blue).

The root mean square deviation (RMSD) of the protein backbone during the simulation against the backbone of the starting structure shows a different behavior for the system at pH 4.0 with respect to the other systems. A mean value of ∼3.5 Å for the simulation at pH 7.0 and of ∼2 Å for the simulation at pH 10.0 is reached in less than 1.0 ns and is maintained during the simulation. On the contrary, at pH 4.0 the system seems not to reach an equilibrium mean value of RMSD (Figure 6A). This different behavior suggests that at pH 4.0 the protein is kinetically more unstable than in the other two pH conditions. These data correlate with the results of the calculations of the diffusion coefficient at this pH value. Moreover, the system appears to be more stabilized at pH 10.0 than at pH 7.0, since the RMSD of the backbone of the protein at neutral pH is higher than the one at basic pH values.

We analyzed also the clusters of similar structures present in the trajectories. We found that at pH 7.0 and 10.0 there is essentially a structure that prevails along the entire simulation. At pH 7.0 there is a cluster formed by ∼4900 elements; the second one is formed by ∼100 elements and is present only in the first phase (<1 ns) of the simulation. At pH 10.0 it is possible to see a unique cluster of 4990 elements, and few structures are dispersed during the trajectory. The RMS distribution of these structures (Figure 6B) shows a Gaussian distribution with a narrow peak at 0.2 nm for the structures at pH 7.0, indicating that the structures composing the cluster are very similar to each others (a very small peak around 0.35 nm is probably formed by the structures belonging to the small cluster in the early phase of simulations). At pH 10.0 the RMS distribution is still a Gaussian distribution, but more enlarged and with some shoulders, indicating that there are probably distinct subpopulations of structures in the cluster, but the differences are below the threshold fixed to cluster the structures. Therefore, it is possible to infer that the conformational variability of the protein in these two conditions is minimal at pH 7.0, more evident at pH 10.0. On the contrary, at pH 4.0 it is possible to note the presence of at least two distinct clusters of structures up to 25 ns of simulation. In the second half of the trajectory, another further cluster is highlighted in the analysis. The main cluster is formed by ∼4700 elements, whereas the other two clusters are formed by 200 and 100 structures, respectively; however, they are coexisting during the entire trajectory. Moreover, the RMS distribution of the structures shows a curve formed clearly by at least two different main components, and other minor components enlarging the Gaussian distribution. From all these data it seems that at acidic pH, MalE2 shows an enhanced instability of its tertiary structure. In a previous paper we correlated the presence of multiple clusters of structures in the MD trajectory with the presence of a molten globule state of the protein [52]. The present situation seems to be similar to that one, suggesting that even if the timescale of simulation is not sufficient to follow the complete unfolding of the protein, it can be sufficient to see the early signal of relaxation of the tertiary structure, anticipating the protein unfolding. Moreover, the impossibility to calculate a meaningful diffusion coefficient for the trajectory at pH 4.0 could be due precisely to the coexistence of multiple tertiary conformations of the protein during the simulation. This fact therefore further supports the hypothesis that at pH 4.0 the protein is forming a transition state that then evolves into unfolding.

**Figure 7:**
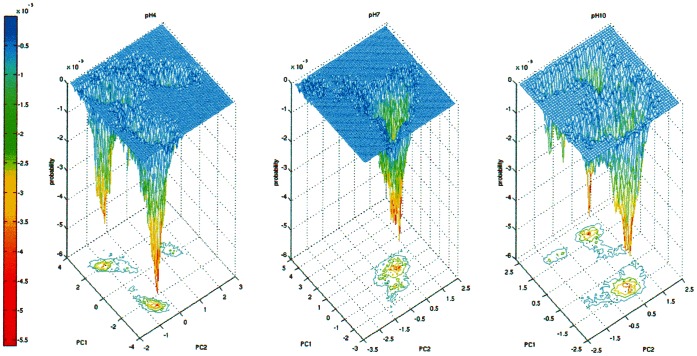
Free energy landscape of MalE2. Projection of the trajectories of MalE2 at pH 4.0 (panel A), pH 7.0 (panel B) and pH 10.0 (panel C) in the essential plane defined by the two first eigenvectors calculated for the simulations. The probability is expressed as relative to the maximum frequency. The color scale (blue-red) defines the most probable conformation.

Going down into the levels of protein structures, the analysis of the variation of secondary structures of the three systems made by DSSP shows that they are practically not influenced by the pH, since the content in secondary structures is nearly identical in the three conditions and is stable during the time of simulation. Also this result is in excellent agreement with far-UV CD data (data not shown). Therefore, it is confirmed also at molecular level that the perturbation produced by pH acts essentially on the overall tertiary fold of the protein. In order to gain insight into the protein flexibility and to identify large scale collective motions of atoms in MD simulations we performed a PCA on the three trajectories and identified the eigenvectors of the mass-weighted covariance matrix of the atomic positional fluctuations calculated on protein Cα atoms [38]. The results of PCA analysis suggests that probably no clear-cut collective motions are present during the simulation. The regions of the protein mainly affected by the motions described by the first two eigenvectors are the segments 25–50 and the segments 305–325 for simulations at pH 4.0, and the regions near residue 100 and 170 for simulations at pH 7.0 and pH 10.0. For each simulation at different pH, the results of PCA analysis were used to represent the free energy landscape (FEL) by projecting the MD trajectory on the essential plane defined by the first two eigenvectors (PC1 and PC2). Figure 7 shows the results of this calculation. At pH 7.0 there is a single hollow corresponding to the most probable conformation of this trajectory. On the contrary, at pH 4.0, at least three conformations are present, two of which with a deeper value of probability and with a narrow hollow. At pH 10.0 there are several minima, with two deeper hollows, less narrow than at pH 4.0. In conclusion, both analyses at pH 4.0 and pH 10.0 indicate the presence of multiple protein conformations probably due to the partial the unfolding of the MalE2 tertiary structure as a consequence of the induced perturbation of native ionic interactions.

### Conclusions

In recent years, the development of novel techniques for the detection and analysis of single molecules has opened up a new era of biological research. In contrast to ensemble methods, which only yield average values for physical and chemical properties and parameters, single-molecule experiments provide information on distributions and time trajectories that would otherwise be hidden by the statistical mean. Using MalE2 as a model, this work demonstrates that new, important insights on the proteins dynamical features can be nicely obtained integrating two different and powerful methodologies, such as FCS and MD.
